# Long-Acting Reversible Contraception: Placement, Continuation, and Removal Rates at an Inner-City Academic Medical Center Clinic

**DOI:** 10.3390/jcm10091918

**Published:** 2021-04-28

**Authors:** Aliye Runyan, Robert A. Welch, Katherine J. Kramer, Sarah Cortez, LeAnne J. Roberts, Clementina Asamoah, Sarah Ottum, Jessica Sanders, Adib Shafi, Maurice-Andre Recanati

**Affiliations:** 1 Department of Obstetrics and Gynecology, Westchester Medical Center, Valhalla, NY 10595, USA; alrunyan@gmail.com; 2 Department of Obstetrics and Gynecology, Hurley Medical Center, Flint, MI 48503, USA; rwelch2@hurleymc.com; 3 Department of Obstetrics and Gynecology, St. Vincent’s Catholic Medical Centers, New York, NY 10011, USA; katherinekramer@gmail.com; 4 Department of Obstetrics and Gynecology, Wayne State University, Detroit, MI 48202, USA; sarah.cortez@wayne.edu (S.C.); lroberts@med.wayne.edu (L.J.R.); asamoahc@gmail.com (C.A.); 5 Department of Surgery, Wayne State University, Detroit, MI 48202, USA; Sottum@med.wayne.edu; 6 Department of Obstetrics and Gynecology, University of Utah, Salt Lake City, UT 84132, USA; jessica.sanders@hsc.utah.edu; 7 Department of Computer Science, Wayne State University, Detroit, MI 48202, USA; fj9079@wayne.edu; 8 NIH-Women’s Reproductive Health Research (WRHR) Scholar, Department of Obstetrics and Gynecology, Wayne State University, Detroit, MI 48202, USA

**Keywords:** long-acting reversible contraception, intrauterine device, LARC placement delays, removal rates, inner-city clinic

## Abstract

Long-Acting Reversible Contraception (LARCs) has the potential to decrease unintended pregnancies but only if women can easily access a requested method. Retrospective electronic chart review identified women desiring LARC placement over a one-year period ending 31 December 2016. Most of the 311 insertions were for family planning, with 220 new insertions and 60 replacements. Delays occurred in 38% (*n* = 118) of patients, averaged 5 ± 5 weeks, and 47% received interval contraception. Reasons included absence of qualified provider (*n* = 44, 37%), pending cultures (*n* = 31, 26%), and Mirena availability. Teenage LARC use favored Nexplanon whereas older women preferred Mirena (*p* < 0.01). Of the 11% choosing early LARC removal, a significant number were African Americans (*p* = 0.040) or teenagers (*p* = 0.048). Retention time varied by device type; most patients switched to other contraceptives. No patients experienced IUD expulsion. Understanding barriers, attempting to remedy them, and addressing the side effects associated with LARC use is of importance in this inner-city patient population in the United States.

## 1. Introduction

About six million pregnancies occur yearly in the United States, with approximately 45–51% of these unintended [1]. Compared to other industrialized nations, this rate is substantially higher [2], and worldwide 44% of pregnancies are unintended, with rates as high as 65% in developing nations [3]. Although unintended pregnancies affect all women, rates were disproportionately highest among those who were cohabitating, below the poverty line or were non-Hispanic Blacks [1]. About 54% experiencing unintended pregnancy reported using contraception at the time they conceived [4]. Inconsistent method use accounts for 90% of these pregnancies rather than method failure (10%) [5]. About 25% of at-risk women experience one or more months with a gap in contraceptive use [6]. Lack of college education, ambivalence about pregnancy, being Black, being 35–44 years of age, method-related difficulties, side effects and dissatisfaction with the current method were strongly associated with inconsistent contraceptive use [7].

Of the 61 million U.S. women aged 14–44 years old, 61.7% are currently using contraception [8]. Common methods include oral contraceptives (16–38%), male condoms (9.4–32%), long-acting reversible contraceptives (LARC) (7.2–12%), and female sterilization (15.5–25%) [5,9]. These rates are comparable with other nations such as Europe (10–32%) and Australia (7%) [10]. Failure rates in the first year are 9%, 18%, 0.05–0.8%, and 0.1–0.8%, respectively [11]. Reasons for inconsistent use of user-dependent methods include absence from home, depletion of supplies, illness, lack of inclination, perception of being at low risk for pregnancy, attitudes about pregnancy, and experiences with unintended pregnancy [4,5].

LARC methods of contraception are highly effective yet may be underused [11]. Only about 12% of women currently employ LARC contraception, of whom 10% use intrauterine devices (IUDs) while 1.3% have subdermal implants (SDIs) [9]. Barriers to LARC utilization include patient lack of knowledge [12], bias in counselling by healthcare providers [13,14], lack of provider training and competency [15], and the up-front cost of these devices [16]. When patients are well educated, and when costs are not a factor, such as for female OBGYN residents, 53% chose LARC methods [17]. Similarly, for patients enrolled in the CHOICE Project, which provided education and no-cost birth control, a majority of women (67%) choose LARCs [18]. Because LARC methods are “forgettable,” effectiveness is user-independent, and they eliminate the common shortfalls previously described for other contraceptives. LARCs have some of the highest rates of continuation after one year—84% for Implanon [11] and greater than 80% for IUDs [11]—with patient satisfaction rates mirroring continuation rates [19,20]. These devices have been shown to be safe [21] and effective in all women regardless of parity [22] and their use has been increasing in younger women [23].

In order to prevent unintended pregnancies and their associated financial [24] and social [25] costs, effective contraceptives, such as LARCs, must be readily accessible and barriers to insertion minimized. Since nearly half of patients intending to use an IUD failed to return for placement during a subsequent visit [26], single visit protocols ensure that more women have access to contraception in a timely manner. The insurance restriction of combining a well-woman exam with IUD insertion, the availability of the device, and pre-insertion screening all may cause such delays. Multiple studies have shown that in women with asymptomatic sexually transmitted diseases, IUD placement did not increase the risk of pelvic inflammatory disease (PID) [27,28]. Thus, screening can be done at the time of insertion and treatment given subsequently [29].

For a contraceptive to be effective it must be continuously used, yet a minority of patients opt to discontinue their method for reasons other than planning pregnancy [30]. Common adverse effects of LARCs include abnormal bleeding, amenorrhea, dysmenorrhea, pelvic pain [31] and, for implants, rod migration, mood swings, and headaches [32]. While LARCs are FDA approved to remain in place for 3 to 10 years, women who experience intolerable side effects may opt for “early” removal [33,34,35]. Many patients requesting removal of LARCs also complained of a lack of disclosure of all possible side effects and encountered delays after they requested removal, raising concerns about provider’s attitudes and respect for body autonomy [36,37].

LARC utilization patterns at the downtown campus of the Detroit Medical Center are likely to reflect those of other large urban populations. Little is known, however, about LARC acceptance by underserved women in the impersonal environment of such large urban medical centers that are staffed by resident physicians and nurse practitioners. The impact of high-volume clinic care on the acceptance of LARCs may have significant population health implications. The purpose of this study was to evaluate LARC utilization in the setting of a large urban academic center.

## 2. Materials and Methods

### Patient Selection

Under IRB approval (WSU 074517MP2E), a retrospective electronic health record (EHR) review was performed to identify women who requested LARC placement in the resident clinic for a one-year period from 1 January through 31 December 2016. Included were non-pregnant women [38] desiring LARC contraception. IUDs (Mirena and Paragard) and implants (Nexplanon) were analyzed separately. Information from each patient included: demographic data, prior contraceptive method, proximity to previous childbirth, date of LARC request, date of LARC placement, type of device, provider (resident, physician or nurse practitioner), reason device was inserted (e.g., family planning, replace expiring device, abnormal bleeding) and an explanation for delays, defined as failure to place a device at the initial visit. Records were reviewed for prescription of bridging contraceptive methods during this delay period. Patients receiving LARCs during the study period were followed for an additional year to identify those requesting early LARC removal, the date of this request, reason, and actual date the device was removed, as well as their new contraceptive method, if any.

Quantile-quantile plots were used to examine normality of numeric variables. Median differences were examined using the Kruskal–Wallis or Mann–Whitney U tests, as appropriate. Differences in proportions were tested using the Fisher’s exact test, as appropriate. We used logistic regression to examine magnitudes of association adjusting for potentially confounding factors. Statistical significance was considered to be *p* < 0.05 or 95% confidence interval (CI), not including the null estimate of association (i.e., odds ratio (OR) = 1.0).

## 3. Results

This study included 311 participants. LARC device utilization by age and ethnicity is shown in Table 1.

### 3.1. Indication for Device Placement

The majority of LARCs were placed for family planning indications (*n* = 280) with *n* = 220 new device insertions and *n* = 60 replacement of an expired device. A significant number of new devices placed included Nexplanon and Paragard (*n* = 87, *p* < 0.004; and *n* = 43, *p* < 0.001, respectively), compared to all insertions. A significant number of new family planning insertions were requested by Middle Eastern women (*n* = 17, *p* = 0.014), compared to all insertions for the indication of family planning. Abnormal uterine bleeding accounted for the remainder of insertions (*n* = 31), all of which were Mirena.

### 3.2. Device Distribution by Race

**Figure 1:**
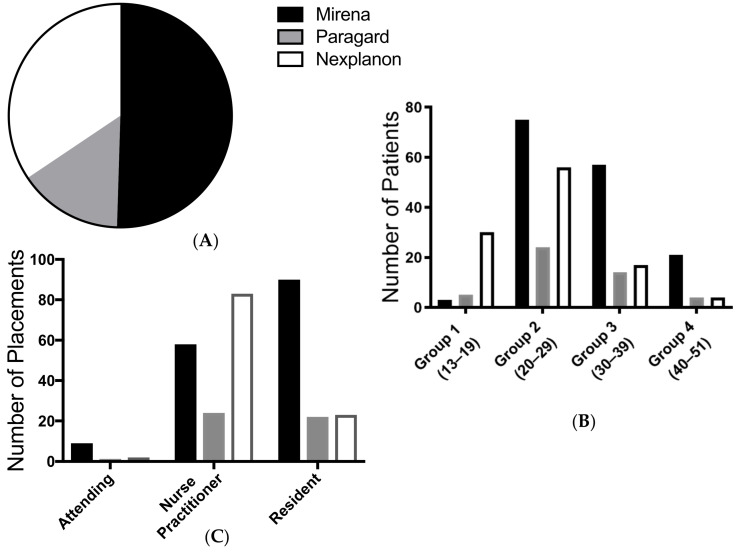
LARC devices placed in 2016 (**A**) by device type (*n* = 311); (**B**) by age group; (**C**) by provider type.

Across all cases (new insertions and replacements), Mirena was the most frequently chosen LARC (*n* = 157, 50%), followed by Nexplanon (*n* = 107, 34%) and Paragard (*n* = 47, 15%) as shown in Figure 1. African Americans chose Mirena significantly more than did the other races (Middle Eastern, Asian, White and Hispanic), 55% vs. 24% (146/266 vs. 11/45, *p* < 0.001). Similarly, African Americans chose Nexplanon significantly more often than did the other races, 38% vs. 11% (102/266 vs. 5/45, *p* < 0.001). On the other hand, the other groups, particularly Middle Eastern, chose Paragard significantly more than did African Americans, 64% vs. just 7% (29/45 vs. 18/266, *p* < 0.001).

### 3.3. Device Distribution by Age

For analysis purposes, age ranges were divided into four groups: group 1: 13–19 years old (*n* = 38), group 2: 20–29 (*n* = 155), group 3: 30–39 (*n* = 89), group 4: 40–51 (*n* = 29). A significant number of patients from group 4 chose Mirena (*n* = 21, *p* = 0.01), while a significant number of teenagers chose Nexplanon (*n* = 30, *p* < 0.001), as shown in Figure 1. 

### 3.4. Analysis of Delays in LARC Placement

Out of 311 patients, 38% (118) experienced delays requiring more than one visit for the placement of their LARC, and the mean length of the delay was 4.8 weeks ± 4.8 (SD, range 1 week to 7 months).

African American women were significantly more likely to experience a delay in LARC placement compared to other races taken as a whole (108/266 = 41% vs. 10/45 = 22%, *p* = 0.020). There was no significant difference, however, in the duration of delay by race.

Those choosing Mirena were significantly more likely to experience a delay in LARC placement compared to the other two devices taken as a whole (69/157 = 59% vs. 49/154 = 32%, *p* = 0.035). There was no significant difference, however, in the duration of delay by device.

**Figure 2:**
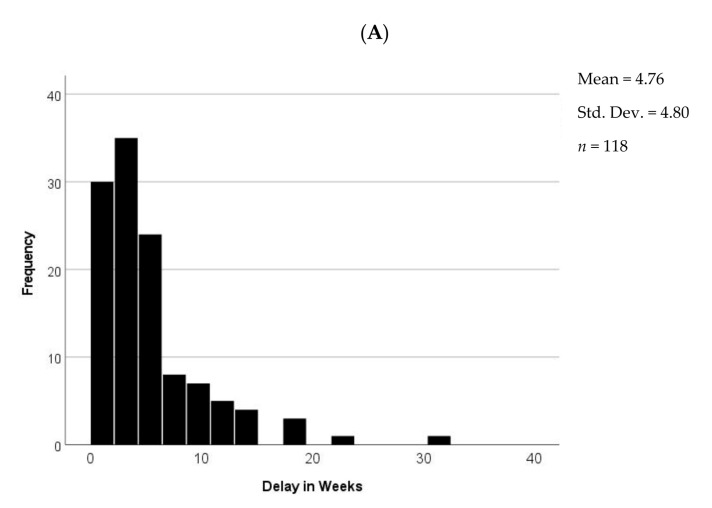

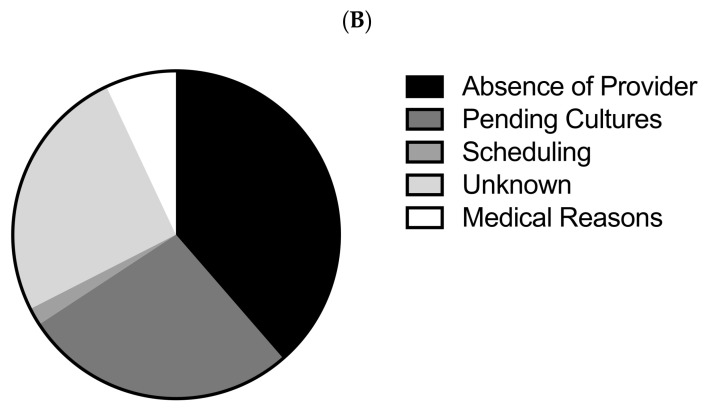
Delay in LARC Placement. (**A**) Length of delay; (**B**) reasons for delay.

Stated reasons for delaying LARC placement included absence of a qualified provider (*n* = 44, 37%), pending gonorrhea/chlamydial test results (*n* = 31, 26%), and reason unknown (*n* = 30, 25%), with the remainder consisting of scheduling issues (*n* = 2), recent intercourse (*n* = 2), abnormal pap (*n* = 1), unexplained vaginal bleeding (*n* = 1), pending endometrial biopsy (*n* = 4), retained IUD (*n* = 2) and uterine abnormality (*n* = 1) (Figure 2). A significant number of patients who were delayed for pending gonorrhea/chlamydial cultures had chosen Mirena compared to all who were delayed (*p* = 0.007, *n* = 25 of 31 compared to 69 of 118). Patients who chose Nexplanon had significantly less delay in placement (*p* = 0.020) when the device was inserted by residents (*n* = 8 of 35) as opposed to nurse practitioners (*n* = 26 of 35). No other association was found between the delay in LARC placement and the type of medical provider. No significant association was found between patient age and delay in LARC placement. Forty-seven percent of women experiencing delays were bridged with contraceptives including condoms (*n* = 24, 21%) or continuation of existing method (*n* = 29, 26%) such as Depo-Provera or IUD, while the rest were either not offered contraception or no documentation could be found.

### 3.5. Analysis of Device Removal

**Figure 3:**
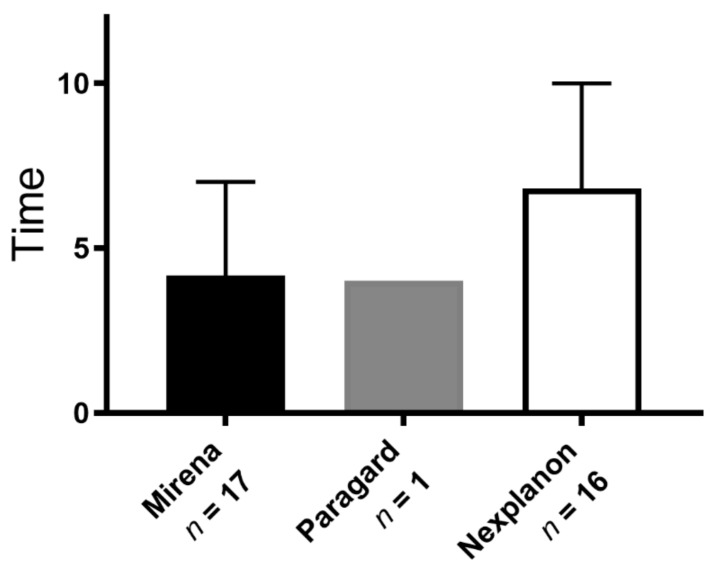
Thirty-four (11%) women chose early removal of their LARC. Mean time (in months) from insertion to removal varied significantly by device type. Error bars represent SD.

Thirty-four women chose to have their LARC device removed (17 Mirena, 16 Nexplanon, 1 Paragard). Out of this group, *n* = 24 (71%) were new insertions. The average length of retention for those who chose removal was 162 days (range 8–356 days) but varied significantly (*p* = 0.02) by device type: Mirena (4.2 months ± 2.8 SD) vs. Nexplanon (6.8 months ± 3.2 SD) (Figure 3). No significant relation was found with the type of provider placing the device. Nearly all the women who chose to remove their LARC were African American (33/34, *p* = 0.040). A significant number who chose removal were teenagers (*n* = 8, *p* = 0.048). Only 3% (1 of 34, *p* = 0.39) discontinued Paragard, despite Paragard comprising 15% of all devices (47 of 311). No patients experienced IUD expulsion.

### 3.6. Analysis of Switching out of LARC Method

Of the 34 women who removed their LARC, twenty-four elected to switch to an alternate method of contraception. These included: OCPs (*n* = 10), depot-medroxyprogesterone acetate (*n* = 9), Paragard (*n* = 1), patch (*n* = 1), Mirena (*n* = 1), NuvaRing (*n* = 1), and barrier methods (*n* = 1). Of all age groups, teenagers were also most likely to switch to another form of birth control (*n* = 8, *p* = 0.018).

## 4. Discussion

### 4.1. Delay in Placement

The majority of LARC placements were for family planning and 21% (60/280) of placements consisted of replacement of an expiring device. Our experience in this urban clinic demonstrates that 38% of patients requesting LARC experienced significant delays in placement. Lack of provider education and particularly the belief that IUD placement requires knowledge of the results of gonorrhea/chlamydia testing [39], the absence of a qualified providers, and scheduling issues accounted for about two-thirds of these delays (Figure 2). Mirena was the most frequently chosen LARC, but women opting for this device experienced the greatest delays due to pending cultures (*p* = 0.022) and because of lack of device availability.

Unintended pregnancy remains a major public health issue [1]. LARC contraceptives, when placed on the day that the patient requests it, provides immediate protection from unintended pregnancy. In women where it is “reasonably certain” [38] that they are not pregnant, same day placement should be the goal [40]. When compared to patients requesting hormonal methods, 95% women (697/737) who met the criteria walked out with a prescription. These factors may be addressed through provider education and improved clinic management. Clinicians should offer bridging contraception to prevent pregnancy regardless of the reason for the delay in LARC placement.

### 4.2. Early Removal of Device

We found that both subdermal implants (SDI) and IUDs had high continuation rates (90%) and low numbers of complications in the first-year post-insertion, consistent with other studies [19,35,41]. These rates surpass those of short-acting contraceptives such as the patch (10%) and the pill (32%) [42]. Unlike other studies [41], we found that removal rates were not associated with type of provider placing the device. This may be due to the formal training clinicians practicing at our institution receive which ensures that patients universally receive adequate pre-placement counselling.

About 10% of LARCs in our study were inserted in teenagers, reflecting a growing acceptance of long-acting contraceptives in this age group [43]. Similar to other studies [33,44], we observed that SDIs were prescribed significantly more in teens. This may be due to an ongoing bias from less informed physicians on the use of IUDs in nulliparous patients [45], and in particularly teens [14]. This group also experienced a significantly higher number of complications, particularly spotting, but were most likely to switch to another form of birth control. Conversely, older women were more likely to opt for an IUD, particularly Mirena, as this method is also indicated for the treatment of abnormal uterine bleeding. Similar to other studies, we found that this age group tended to have high retention rates for their LARC [30]. Future studies may also examine the decision to choose LARCs over other methods where metabolic [46] and vascular effects, weight gain, body image [47] and sexual behavior [48] impact patient wellbeing.

Concerns involving early removal of LARCs go beyond the simple cost-effectiveness calculation and include risks of unintended pregnancies associated with no contraception or suboptimal protection. Frequently, patients present to clinic requesting removal of their device because of pain, changes in bleeding patterns, mood swings, weight gain or other reasons. Studies have shown that these women tended to reluctantly tolerate these side effects but eventually reached a tipping point [32]. A close follow up with patients who received LARCs may help ensure that clinicians can intervene in alleviating the side effects of the LARC. While some physicians tend to be reluctant to remove LARCs, it is critical that they respect patient autonomy and support a woman’s decision-making around contraceptive use [36]. Teaching patients about IUD self-removal [49] could have a positive impact on reproductive autonomy. The effects of women seeking removal from other willing clinicians, or removing the device on their own, may influence the results of our study and will be examined by prospective trials in the future.

A strength of our study is the inclusion of African American women of all age ranges. The study was limited by using a single urban health clinic where the majority of patients had public health insurance [25]. Further study in different geographic areas or in women with private insurance may alter our findings or limit generalizability. If patients received care for LARC-related issues at other clinics, or lost Medicaid eligibility, continuation rates may be falsely high. While the electronic health record (EHR) was used to access contraception encounter data, the accuracy of the records has the potential for incomplete data [50], which may affect results. One such limitation is the inability of our EHR to ascertain the number of women who intended to be on LARC but were unable to obtain it and never returned.

## 5. Conclusions

Although the American College of Obstetrics and Gynecology recommends same-day LARC placement in eligible patients, this study elucidates some of the challenges encountered in making same-day insertion practical in a large urban clinic. Realizing that women requesting family planning visits should be considered urgent appointments can help protect women from undesired pregnancy. Controlling device inventory, having trained providers present and educating on latest guidelines are necessary to fulfill patient expectations. In all circumstances, clinicians must offer the patient a reliable means of contraception in the interim. Reasons why women chose LARC removal and choose other, less reliable forms of contraception need further study.

## Figures and Tables

**Table 1:** Utilization of Long-Acting Reversible Contraception by Age and Ethnicity.

	N (%)	Mirena	Paragard	Nexplanon
Average Age (±SD)	28.2 (±7.9)	30.9 (±7.6)	28.4 (±7.4)	24.1 (±6.8)
Age range	13–51	14–51	17–46	13–44
African American	266 (85)	146	18	102
Middle Eastern	18 (6)	3	14	1
Asian	15 (5)	2	13	0
White	10 (3)	5	2	3
Hispanic	2 (<1)	1	0	1
Family Planning—New device insertions	220 (71)	90	43	87
Family Planning—Replacement of expired device	60 (19)	36	4	20
Abnormal Uterine Bleeding	31 (10)	31	0	0
Total	311 (100)	157	47	107

## Data Availability

The datasets that support the findings of this study are available from the corresponding author (M.-A.R.) upon reasonable written request.
